# Utility of the Tympanic Membrane Pressure Waveform for Non-invasive Estimation of The Intracranial Pressure Waveform

**DOI:** 10.1038/s41598-018-34083-6

**Published:** 2018-10-25

**Authors:** Karen Brastad Evensen, Klaus Paulat, Fabrice Prieur, Sverre Holm, Per Kristian Eide

**Affiliations:** 1 Department of Informatics, University of Oslo, Oslo, Norway; 2 Institute of Medical Engineering and Mechatronics, Hochschule Ulm, Ulm, Germany; 3 Department of Neurosurgery, Oslo University Hospital - Rikshospitalet, Oslo, Norway; 4 Institute of Clinical Medicine, Faculty of Medicine, University of Oslo, Oslo, Norway

## Abstract

Time domain analysis of the intracranial pressure (ICP) waveform provides important information about the intracranial pressure-volume reserve capacity. The aim here was to explore whether the tympanic membrane pressure (TMP) waveform can be used to non-invasively estimate the ICP waveform. Simultaneous invasive ICP and non-invasive TMP signals were measured in a total of 28 individuals who underwent invasive ICP measurements as a part of their clinical work up (surveillance after subarachnoid hemorrhage in 9 individuals and diagnostic for CSF circulation disorders in 19 individuals). For each individual, a transfer function estimate between the invasive ICP and non-invasive TMP signals was established in order to explore the potential of the method. To validate the results, ICP waveform parameters including the mean wave amplitude (MWA) were computed in the time domain for both the ICP estimates and the invasively measured ICP. The patient-specific non-invasive ICP signals predicted MWA rather satisfactorily in 4/28 individuals (14%). In these four patients the differences between original and estimated MWA were <1.0 mmHg in more than 50% of observations, and <0.5 mmHg in more than 20% of observations. The study further disclosed that the cochlear aqueduct worked as a physical lowpass filter.

## Introduction

Continuous monitoring of intracranial pressure (ICP) is routine in everyday neuro-intensive care, and is an invaluable tool in the surveillance of critically ill patients^1^. Timely detection of raised ICP allows for early management which reduces the risk of permanent brain damage, and improves patients outcome after brain injury caused by e.g. trauma or stroke^2,3^.

Currently, measurements of ICP require a neurosurgeon to drill a hole in the patient’s skull and advance a catheter into the brain parenchyma, or through the brain tissue and into the ventricular space. This is an invasive procedure, associated with risk of severe complications such as infection and hemorrhage in about 1–2% of patients^4^. Despite a 40-year history of research on non-invasive ICP, none of the presented methods are currently accurate enough to be used in clinical practice^5–8^. Therefore, ICP monitoring is restricted to patients with severe brain disease, in whom the invasiveness of the procedure is outweighed by the importance of measuring ICP.

As ICP monitoring would benefit a significantly larger patient pool, there has been a considerable effort among neuroscientists to find alternative measures to evaluate the brain’s condition and anticipate brain deterioration^9^. One of the suggested measures is the intracranial pressure-volume reserve capacity, often referred to as the intracranial compliance (ICC), which is given as the relationship between the change in volume and change in pressure dV/dP^10^. If the intracranial system has high compliance, a small increase in volume, caused by for example a cerebral hemorrhage, will result in a small pressure increase. If the system has low compliance, the same volume increase will result in a much higher intracranial pressure increase. Knowledge about the ICC of the system would therefore give the clinicians important information about the patient’s condition.

While ICP usually is assessed by the mean ICP, representing the absolute pressure difference between the outside and inside of the skull cavity, an increasing body of data suggests that the intracranial pressure-volume reserve capacity is better described by the ICP waveform than the mean ICP itself^11–15^. One key component of the ICP waveform is the time averaged peak-to-peak value, here denoted Mean Wave Amplitude (MWA), which can be considered as a surrogate marker of ICC^13,16^. Hence, clinical studies show that managing patients with intracranial bleeds (subarachnoid hemorrhage, SAH) according to MWA gave better outcome than management according to the traditional mean ICP^17^. Therefore, Non-invasive techniques for monitoring the ICP waveform, including waveform parameters such as MWA, could have significant clinical value.

One suggested technique for non-invasive monitoring of the intracranial pressure-volume reserve (compliance) is to utilize the pathway from the intracranial compartment to the inner ear named the cochlear aqueduct. This approach was first proposed by Marchbanks in the late 1970s, who reported that patients with raised and normal ICP showed differences in tympanic membrane displacement (TMD) in response to the stimulation of the stapedial reflex^18^. The technique was later commercialized, and this equipment has been used to measure absolute, or mean ICP, in several different studies^19–21^. The study by Gwer *et al*.^20^ found TMD measurements to give indications of raised mean ICP, but in the study by Shimbles *et al*.^19^ the technique was applied successfully to only 40% of the patient population. In general, the inter-subject variability was found to be too large for clinical use. In a study by Davids *et al*. it was suggested that the pulse shaped waves possible to measure using the Marchbanks system were generated by ICP waves transmitted through the ossicular chain^22^. The authors were able to change the ICP level by varying the subject’s head position, giving raise to different outer ear pressure waveforms in the supine and sitting positions, suggesting that difference in pressure waveforms could give information about the intracranial compliance.

The aim of the present study was to investigate whether the pathway between the inner ear and the intracranial compartment can be used to non-invasively predict the peak to peak amplitude of the ICP waveform, here used as an indication of intracranial compliance. The hypothesis is that the infrasonic ICP waves propagate through the cochlear aqueduct, the inner ear and middle ear, and are possible to measure in the outer ear due to the movement they cause in the tympanic membrane. If so, this would provide additional clinical information currently not assessable with traditional TMD measurements and without the uncomfortable stimulation of the stapedial reflex.

## Methods

### Ethical permissions

The study was approved by The Regional Committee for Medical and Health Research Ethics (REK) of Health Region South-East, Norway (approval no. 2005/4307), and was performed in accordance with relevant guidelines and regulations. Patients were included after oral and written informed consent.

### Patients

In order to evaluate the hypothesis, the study enrolled individuals undergoing continuous ICP monitoring as part of surveillance or diagnostic assessment of at the Department of Neurosurgery, Oslo University Hospital-Rikshospitalet.

### Rationale behind TMP measurements

**Figure 1:**
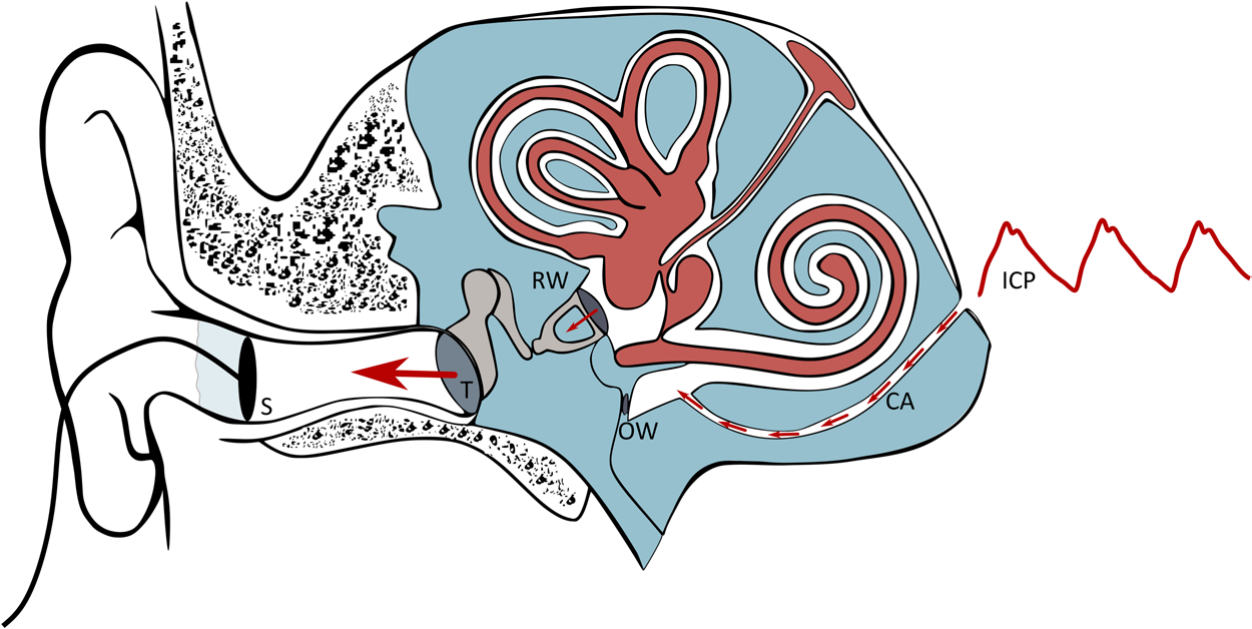
Schematic illustration of the anatomical structures involved in measurements of TMP waveforms. In this study, non-invasive TMP waveforms were measured in the outer ear and used as input for the estimation of non-invasive ICP. The labels represents the following; ICP: ICP input signal, CA: Cochlear Aqueduct, OW: Oval Window, RW: Round Window, T: Tympanic membrane and S: Sensor.

Figure 1 provides an overview of the anatomical structures involved in the TMP measurements. The perilymph of the inner ear primarily communicates with the cerebrospinal fluid (CSF) space of the posterior cranial fossa via a bony canal called the cochlear aqueduct. Studies done in monkeys/guinea pigs^23^ and cats^24–26^ show that a patent cochlear aqueduct allows for pressure transfer between the perilymphatic fluid and the cerebrospinal fluid. The ICP waveforms have the majority of their energy below the 20 Hz hearing threshold and will propagate via the non-compressible fluid in the cochlear aqueduct to the perilymphatic space of the inner ear. Here the infrasonic waves cause motion of the oval window, the ossicles, and thereafter the tympanic membrane, as illustrated in Fig. 1. If the outer ear is sealed completely, so that the ear canal is perfectly airtight, the oscillations of the tympanic membrane should be possible to detect in the outer ear using a very high-resolution transducer. The goal is hence to do non-invasive pressure waveform measurements in the outer ear, and use these to estimate the cochlear fluid pressure waveform, which potentially can be a measure of ICP^27,28^.

### Non-invasive TMP waveform measurements

**Figure 2:**
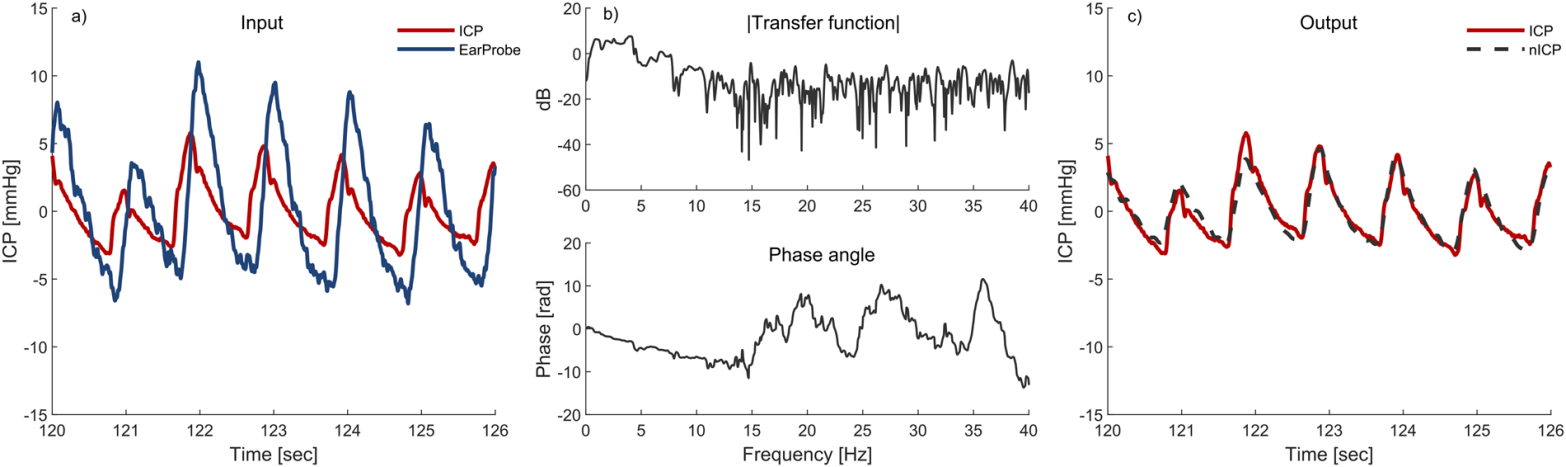
An example showing 6 seconds of the input signals and the corresponding transfer function estimate based on a total 10 minutes is shown in Figure (**a**,**b**) respectively (patient ID 3). The resulting output from the combination of Fig. 1a and the inverse of Fig. 1b is shown in Fig. 1c.

The non-invasive TMP signals were measured from the outer ear channel using a specially designed pressure transducer developed by Paulat and coworkers^29^. The sensor is a highly sensitive pressure sensor designed to log the movement of the tympanic membrane, i.e. the change in pressure difference relative to the ambient air pressure. In order to only measure the motions of the tympanic membrane caused by the pulsatile ICP wave, and not external pressure fluctuations, the ear canal was completely sealed. This was done by placing a tube in the outer ear canal which was closed air-tight using a glove around the tube within the outer ear canal. This is the same equipment applied by the Otorhinolaryngology Department when fitting hearing aids. The tympanic membrane pressure waveform was thereafter measured continuously for 0.5 to 5 hours with measurement time varying from patient to patient. A sampling frequency of 200 Hz was used to secure no loss of time- and frequency domain information^30^. For more details about the measurement procedure and setup, see the relevant technical note^14^. All TMP measurements were done in the right ear, unless there were obvious wounds or other obstructions of the ear canal in this ear. If so, the left ear was used. Both ears were never included. For an illustration of the measurement set up and underlying concept, see Fig. 1. For an example of TMP waveform measurements see Fig. 2a).

### Invasive ICP waveform measurements

Simultaneously with the TMP measurements, continuous measurements of invasive ICP were done with the same time reference and sampling frequency. A small burr hole was made in the skull and a Codman MicroSensor^TM^ (Johnson & Johnson, Raynham, MA, USA) was placed 1–2 cm into the frontal brain parenchyma. Continuous pressure time series were stored as anonymous raw data files for future analysis. For more detailed description of the monitoring procedure for invasive ICP see^31^. The entire measurement time was used in the analysis, with all the fluctuations in ICP level which that entails. The ICP measurements done for diagnostic purpose were done in awake unventilated patients, while individuals undergoing ICP monitoring as part of surveillance were on artificial ventilation (Table 1).

**Table 1:** Demographic information and number of observations.

PatID	Sex	Age (yrs)	BMI (kg/m2)	Reason for ICP	Admission diagnosis
1	F	54	20.4	D	Secondary HC (after SAH)
2	M	59		D	Secondary HC (after TBI)
3	M	71	20.6	D	iNPH
4	M	34		S	Surveillance for SAH
5	F	58	20.7	D	Secondary HC (after SAH)
6	F	53		S	Surveillance for SAH
7	M	60	31.0	D	iNPH
8	F	17	21.9	D	Secondary HC (cerebral tumour)
9	F	75	22.3	D	iNPH
10	M	58		D	iNPH
11	F	27	20.0	D	Communicating HC
12	F	70		S	Surveillance for SAH
13	F	42	22.5	D	Communicating HC
14	F	50		S	Surveillance for SAH
15	M	50	18.6	S	Surveillance for SAH
16	F	42	28.1	D	IIH
17	F	38		D	Communicating HC
18	F	70	27.1	D	iNPH
19	F	48	31.9	D	IIH
20	F	67		S	Surveillance for SAH
21	M	77		S	Surveillance for SAH
22	F	49	20.4	S	Surveillance for SAH
23	F	55	30.1	D	iNPH
24	M	66		D	iNPH
25	M	59	24.3	S	Surveillance for SAH
26	M	73	29.3	D	iNPH
27	F	81	28.9	D	iNPH
28	F	11	29.6	D	Secondary HC (cerebral tumour)
Mean ± std		54 ± 18	25 ± 5		Admission diagnosis

D: Diagnostic. HC: Hydrocephalus. IIH: idiopathic intracranial hypertension. iNPH: idiopathic normal pressure hydrocephalus. SAH: subarachnoid hemorrhage, S: Surveillance.

### Transfer function estimation

When studying the path of the pressure wave propagating from the subarachnoid space to the outer ear, Gopen *et al*.^32^ found that the cochlear aqueduct is the factor that primarily affects the frequencies below 20 Hz. If the cochlear aqueduct is the main component that affects infrasonic frequencies, there should be a relatively simple relationship connecting the inner ear and the subarachnoid space which should be possible to estimate using signal processing.

A linear, time-invariant system is assumed, where the output *y* is connected to the input *x* as $y=h\ast x$. In this relation, *h* is the system’s impulse response, and $\ast$ the symbol for convolution. The Fourier transform of the impulse response gives the transfer function *H*(*f*), which is a description of the system in the frequency domain

1 $$H(f)=\frac{Y(f)}{X(f)},$$

where *H*(*f*) is the Fourier transform of the input signal, and *Y*(*f*) the Fourier transform of the output signal. An estimate of the transfer function $\hat{H}(f)$ can be found from the relation.

2 $$\hat{H}(f)=\frac{{\hat{P}}_{xy}(f)}{{\hat{P}}_{xx}(f)}.$$

In Equation (2) ${\hat{P}}_{xy}(f)$ is the cross power spectral density of the input and output signal *x* and *y*, and ${\hat{P}}_{xx}(f)$ is the power spectral density of the input signal.

In this study, the aim is to establish transfer function estimates for each patient, and thereafter use them to estimate the ICP waveform non-invasively. This is similar as to what is done by Evensen *et al*. in^33^, where estimates of the central aortic wave form have been used as a source signal. In this study however, the time series of the measured ICP signals, here denoted as $x(n),n=0\ldots ,\,N-1$ acts as the source for the corresponding measured TMP signals, which are the output *y*(*n*). Because we have a finite number of samples *N*, the power spectral densities are not known and must be estimated. One possible technique, and the one applied here, is Welch’s averaged, modified periodogram method of spectral estimation. This allows for the signal to be divided into *K* overlapping sequences of length *L* that overlaps by *L* − *D* points. Each sequence is weighted by a window *w*(*n*) and an estimate for the power spectral density for the input signal *x*(*n*) is given in^34^ as

3 $${\hat{P}}_{xx}(f)=\frac{1}{KLU}{\sum _{i=0}^{K-1}|\sum _{n=0}^{L-1}w(n)x(n+iD){e}^{jn2\pi f}|}^{2},$$

where

4 $$U=\frac{1}{L}\sum _{n=0}^{L-1}{|w(n)|}^{2}.$$

The cross power spectral density estimate for the input and output is then given as

5 $${\hat{P}}_{xy}(f)=\frac{1}{KLU}{\sum _{i=0}^{K-1}(\sum _{n=0}^{L-1}w(n)x(n+iD){e}^{jn2\pi f})}^{\ast }(\sum _{n=0}^{L-1}w(n)y(n+iD){e}^{jn2\pi f}).$$

### Non-invasive ICP estimation

The invasive ICP and non-invasive TMP signals were first lowpass filtered in order to remove non-physiological high frequency noise such as the 50 Hz power line hum. The low pass filtering was done using a linear phase FIR (Finite Impulse Response) filter with filter order 104, cut-off frequency of 40 Hz and an attenuation of 96 dB at 50 Hz. The transient was removed from the analysis.

To investigate the method’s potential, a patient specific transfer function estimate was found for each patient, using MATLAB’s predefined *tfestimate* function with a Hamming window (MATLAB and Statistics Toolbox Release 2016a, The MathWorks Inc., Natick, Massachusetts, USA). The first ten minutes of each measurements series were used for the transfer function estimation. These ten minutes were divided into 6-second windows with 50% overlap in order to find a representative average. The mean was removed from the input and output series for the transfer function analysis not to be influenced by fluctuations in the mean ICP level, and in order to avoid side lobe leakage^35^. For patients where several measurement series had been done, the longest series was chosen. After establishing patient specific transfer function estimates, these were applied to the entire patient’s recording. This resulted in non-invasive ICP estimates for each patient based on invasive training data.

An example showing 6 seconds of the input signals and the corresponding transfer function estimate based on a total of 10 minutes for one patient is shown in Fig. 2a) and b) respectively. As we wish to use the TMP signals to estimate the ICP signals non-invasively, we apply the non-causal inverse of the transfer function estimate on the recorded TMP signals. The resulting output from the combination of Fig. 2a) and the inverse of Fig. 2b) is shown in Fig. 2c).

### Transfer function estimate evaluation

The transfer function shown in Fig. 2b) is a representative example from the cohort. When visually inspecting the phase, shown in the lower half of the figure, it becomes evident that it is only linear up to approximately 11 Hz for this patient. A single frequency of the ICP signal $\cos (\omega t)$, which is delayed by a time *τ* by passing through the cochlear aqueduct, will be $\cos (\omega (t-\tau ))$ at the exit. Now, a time-delayed cosine can be written in terms of phase as $\cos (\omega t+\varphi )$. That means that the time delay and the phase delay are related as $\varphi =-\omega \tau$. A phase that varies linearly with frequency is therefore equivalent to a time delay which is constant and does not vary with frequency. If that is the case, the canal is dispersion-less. Assuming this, and that deviations from a linear phase are due to estimation problems due to noise, and not the physics of the canal, the transfer function was limited in frequency to only the linear phase part. A filter was applied to each patient’s nICP estimates and ICP measurements as a secondary study. The filter applied is a frequency sampling filter which is specified in the Fourier domain. In its simplest version, it consists of 1’s in the passband and 0′s in the stopband. However, this introduces unnecessary large ripple in the passband and poor stopband suppression near the transition band. In order to reduce these undesirable effects, one simple method is to introduce one or more samples in the transition band with values between 0 and 1^36^. Here, the simplest possible option is used with only a single transition sample with value 0.5. The limit frequency of linear phase was found for each patient and chosen by visual inspection.

### Comparison of measured and estimated ICP waveforms

To investigate the quality of the non-invasive ICP estimates the unfiltered estimates were compared to the untouched, invasive ICP measurements using a time-domain method currently used in the clinic. The method is described in detail in^31^, and includes identifying the cardiac-induced waves by their beginning and ending diastolic minimum pressure and systolic maximum pressure. For each pulsatile ICP wave, the peak to peak amplitude (dP), rise time (dT) and rise time coefficient (RTC = dP/dT) were found. The averaged values, the mean wave amplitude (MWA), mean wave rise time (MWRT) and mean wave rise time coefficient (MWRTC) were computed for subsequent 6-second (6-sec) time windows. The different parameters, and the averaging, are more thoroughly described in^31^. This analysis was also done for the non-invasive ICP estimates that underwent the described frequency filtering against ICP measurements that had undergone the same filtering.

### Statistics

All statistical analysis was done using SPSS software version 24 (IBM Corporation, Armonk, NY). Statistical significance was accepted at 0.05 level.

## Results Patients

The patient cohort included 28 patients whose demographic information is presented in Table 1. They were enrolled in the study from the period April to August 2005. The reason for ICP monitoring was surveillance after subarachnoid hemorrhage in 9 individuals and diagnostic ICP monitoring for CSF circulation disorders of various causes in 19 individuals (Table 1). The average measurement time was 1 hour and 27 minutes.

### Comparison of ICP estimates and measurements

**Figure 3:**
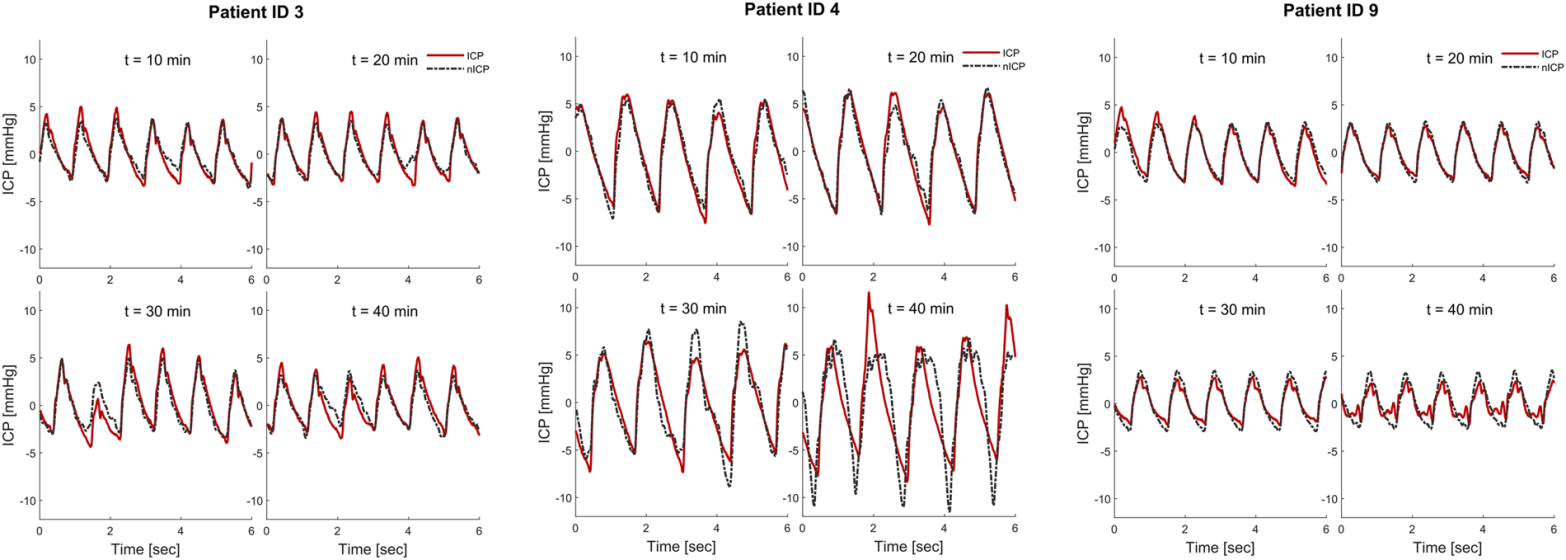
The non-invasive ICP waveform estimate (nICP, interrupted red line) is shown together with the invasive ICP waveform (continuous red line) for four different 6-second time windows after the beginning of the measurement (patient ID 3). The time delay between the nICP and ICP signals, as seen in Fig. 2a, has been removed for visual comparison.

The simultaneous invasive ICP and non-invasive TMP signals included 24,349 6-second time windows from each individual. The invasive ICP scores are presented in Table 2. For each patient a patient specific transfer function estimate was found, which was then used to determine a patient specific ICP estimate. In order to validate the results, the estimated and original ICP waveforms were compared for each individual. An example showing the resulting estimated non-invasive ICP waveform together with the invasively measured ICP waveform for Patient ID 3, 4 and 9 is shown in Fig. 3 for four different time windows. Patient ID 3 was selected because it is a representative example from the cohort, while patient IDs 4 and 9 were chosen as examples of good and poor results, when looking at the average error in MWA prediction.

**Table 2:** Results of estimating pulsatile ICP based on Transfer function from each individual.

PatID	N 6-sec time windows	Original invasive static and pulsatile ICP				Absolute differences between original ICP and estimated non-invasive pulsatile ICP		
		Mean ICP (mmHg)	MWA (mmHg)	MWRT (sec)	MWRTC (mmHg/sec)	MWA (mmHg)	MWRT (sec)	MWRTC (mmHg/sec)
1	462	3.5 ± 1.7	4.2 ± 0.4	0.23 ± 0.02	18.8 ± 1.4	1.6 ± 0.5	0.05 ± 0.03	8.9 ± 2.1
2	666	8.6 ± 3.6	5.3 ± 1.5	0.20 ± 0.04	27.6 ± 7.0	2.3 ± 1.8	0.15 ± 0.05	12.7 ± 8.3
3	409	6.1 ± 1.3	8.0 ± 0.9	0.26 ± 0.01	31.1 ± 3.4	1.6 ± 1.1	0.02 ± 0.02	7.4 ± 4.4
4	1044	33.9 ± 8.6	19.9 ± 7.8	0.31 ± 0.04	64.7 ± 29.6	8.0 ± 6.7	0.07 ± 0.04	32.2 ± 27.2
5	682	13.4 ± 3.7	3.8 ± 0.9	0.25 ± 0.03	15.7 ± 3.2	1.5 ± 1.0	0.11 ± 0.04	7.9 ± 4.7
6	321	6.1 ± 0.9	3.3 ± 0.2	0.19 ± 0.01	18.6 ± 1.0	0.9 ± 0.6	0.02 ± 0.02	6.1 ± 3.0
7	906	21.6 ± 3.5	4.5 ± 0.8	0.11 ± 0.03	8.2 ± 12.8	2.4 ± 0.7	0.10 ± 0.06	35.5 ± 9.8
8	557	0.8 ± 0.8	2.4 ± 0.5	0.12 ± 0.03	23.5 ± 4.4	1.2 ± 0.5	0.16 ± 0.06	19.3 ± 4.8
9	624	2.9 ± 2.2	6.2 ± 0.8	0.29 ± 0.02	21.4 ± 3.8	0.7 ± 0.6	0.04 ± 0.02	4.2 ± 2.9
10	771	16.4 ± 5.6	5.7 ± 1.3	0.21 ± 0.02	27.0 ± 5.0	3.0 ± 1.6	0.11 ± 0.04	17.8 ± 6.9
11	1311	4.9 ± 0.6	3.7 ± 0.3	0.10 ± 0.02	39.7 ± 3.0	1.2 ± 0.6	0.03 ± 0.03	16.9 ± 7.2
12	601	18.1 ± 2.8	7.8 ± 0.5	0.21 ± 0.01	37.7 ± 2.7	3.4 ± 1.1	0.04 ± 0.03	19.8 ± 5.6
13	1134	6.3 ± 1.3	2.4 ± 0.2	0.14 ± 0.04	20.1 ± 3.9	0.8 ± 0.4	0.09 ± 0.04	13.4 ± 4.5
14	239	19.5 ± 2.0	7.2 ± 0.9	0.26 ± 0.03	28.8 ± 4.3	1.8 ± 1.1	0.04 ± 0.03	8.8 ± 4.4
15	750	7.9 ± 3.4	5.6 ± 1.2	0.13 ± 0.05	49.9 ± 11.7	2.4 ± 1.1	0.11 ± 0.06	33.4 ± 12.8
16	524	1.2 ± 1.3	3.4 ± 0.5	0.18 ± 0.05	25.2 ± 7.0	1.2 ± 0.5	0.09 ± 0.05	16.7 ± 6.8
17	227	6.3 ± 1.3	3.0 ± 0.4	0.12 ± 0.03	28.6 ± 4.0	1.2 ± 0.6	0.16 ± 0.05	21.8 ± 4.6
18	2460	13.8 ± 2.9	7.5 ± 1.3	0.24 ± 0.02	32.1 ± 5.1	2.3 ± 1.6	0.07 ± 0.03	11.2 ± 6.8
19	330	9.7 ± 1.6	6.2 ± 0.7	0.18 ± 0.02	35.8 ± 5.0	1.2 ± 0.7	0.05 ± 0.03	12.1 ± 5.7
20	1722	14.1 ± 2.1	7.7 ± 1.3	0.21 ± 0.04	42.0 ± 8.4	3.6 ± 2.2	0.07 ± 0.05	21.1 ± 13.7
21	265	11.7 ± 2.7	13.8 ± 1.9	0.23 ± 0.01	61.7 ± 8.4	3.4 ± 2.4	0.06 ± 0.02	23.1 ± 11.6
22	498	10.7 ± 2.2	4.5 ± 0.6	0.16 ± 0.03	32.9 ± 7.9	2.1 ± 0.8	0.08 ± 0.03	22.7 ± 7.3
23	559	3.3 ± 2.2	3.5 ± 0.5	0.21 ± 0.03	18.5 ± 3.8	1.2 ± 0.5	0.07 ± 0.04	10.3 ± 3.9
24	347	2.7 ± 2.3	6.2 ± 0.8	0.22 ± 0.01	27.7 ± 3.6	1.0 ± 0.8	0.03 ± 0.02	6.3 ± 4.3
25	2957	9.1 ± 2.4	4.3 ± 0.7	0.16 ± 0.03	28.4 ± 4.0	1.6 ± 0.9	0.14 ± 0.05	19.1 ± 6.0
26	420	12.9 ± 2.1	9.0 ± 0.6	0.25 ± 0.01	36.2 ± 2.4	1.7 ± 0.9	0.04 ± 0.02	11.1 ± 3.6
27	2782	8.2 ± 2.0	5.8 ± 1.0	0.26 ± 0.02	22.0 ± 3.8	3.3 ± 1.1	0.10 ± 0.03	15.4 ± 4.2
28	791	42.0 ± 4.7	16.9 ± 4.0	0.17 ± 0.03	103.6 ± 22.2	4.1 ± 3.1	0.07 ± 0.04	36.7 ± 23.3
**AVG** ± **STD**		**11**.**3** ± **9**.**4**	**6**.**5** ± **4**.**2**	**0**.**20** ± **0**.**06**	**34**.**6** ± **18**.**3**	**2**.**2** ± **1**.**5**	**0**.**08** ± **0**.**04**	**16**.**9** ± **9**.**1**
**SUM**	**24**,**359**							

MWA: Mean Wave Amplitude. MWRT: Mean Wave Rise Time, MWRTC: Mean Wave Rise Time Coefficient.

**Figure 4:**
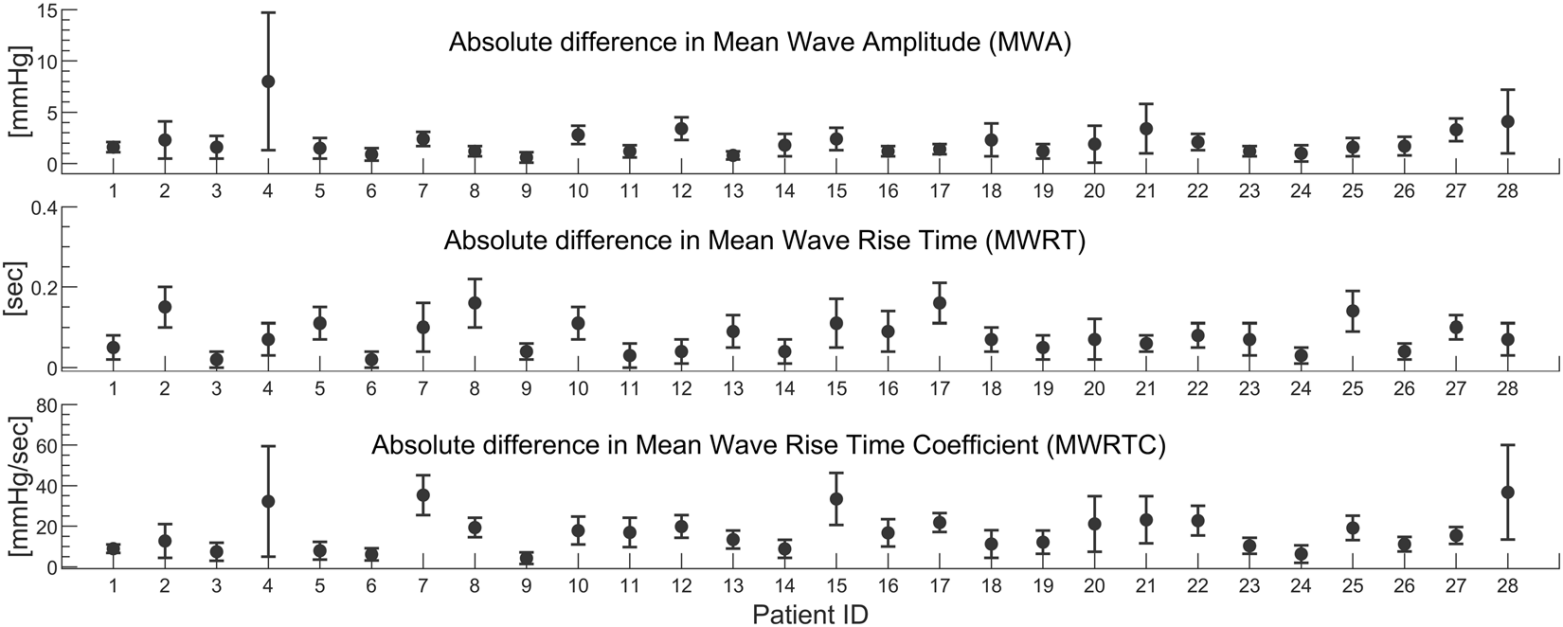
The averaged absolute difference in Mean Wave Amplitude (MWA), Mean Wave Rise Time (MWRT) and Mean Wave Rise Time Coefficient (MWRTC) between the non-invasive ICP estimate and the measured ICP signal is shown for each patient ID. The error bars illustrate the patient specific standard deviation (mean ± stdev).

In clinic, the MWA is most commonly used for diagnostic and surveillance purposes^37,38^. The absolute differences between invasive and estimated ICP scores for the entire measurement for all patients are therefore given in Table 2 and illustrated in Fig. 4. At group level, the absolute difference in MWA between original ICP and estimated non-invasive ICP signals was 2.2 ± 1.5 mmHg (Table 2). This difference was estimated from the entire recording period. When only considering the first 100 6-sec periods, the absolute difference in MWA was 1.7 ± 1.1 mmHg, and when considering the last 10 minutes of recording the absolute difference in MWA was 2.5 ± 1.7 mmHg. Accordingly, the difference in MWA became more pronounced at the end of the measurement period, which indicates lower quality of the TMP signal with time and/or a time varying ICP signal, as the first 10 minutes were used as basis for the transfer function estimation. The same trend becomes evident when comparing the first and last time windows in Fig. 3.

From a clinical perspective, we consider the method to show promise in 4/28 of the individuals. This conclusion was made by looking at the averaged absolute difference between original and estimated MWA. For patient IDs 6, 9, 13 and 24 this was <1.0 mmHg in >50% of observations, and <0.5 mmHg in >20% of observations (Table 3). An average absolute difference of MWA <1 mmHg was observed in 3/28 individuals (Patient IDs 6, 9, and 13; Table 2). In conclusion the non-invasive MWA was estimated rather satisfactorily in four individuals (Patient IDs 6, 9, 13, and 24) (Tables 2 and 3), thus providing a promising non-invasive estimation of MWA in up to 14% of the patient cohort.

**Table 3:** Percentage of 6-s observations with difference in MWA either <0.5 mmHg or <1.0 mmHg.

PatID	Difference in MWA <0.5 mmHg: Percentage of 6-s observations	Difference in MWA <1.0 mmHg: Percentage of 6-s observations
1	1	10
2	10	22
3	14	33
4	10	18
5	17	35
6	23	66
7	2	3
8	4	40
9	45	74
10	11	19
11	9	40
12	2	4
13	23	66
14	14	28
15	5	11
16	15	39
17	12	33
18	12	23
19	12	38
20	9	20
21	5	9
22	0	6
23	5	37
24	33	59
25	13	24
26	3	19
27	1	3
28	8	15
**AVG** ± **STD**	**11**.**4** ± **10**.**0**	**28**.**4** ± **19**.**7**

MWA: Mean Wave Amplitude.

We also examined the ability of the estimates to predict MWA at a threshold of 4 rather than 1 mm Hg, given that this is a commonly used clinical threshold value (Table 4). The negative predictive value for this threshold was low, at only 53% (15/28).

**Table 4:** Predictive ability of MWA threshold of 4 mmHg.

Criteria	Number	Test results
Original & Estimate ≥4 mmHg	13	Sensitivity 65% Specificity 100% PPV 100% NPV 53%
Original & Estimate <4 mmHg	8	
Original ≥4 mmHg & Estimate <4 mmHg	7	
Original <4 mmHg & Estimate ≥4 mmHg	0	

PPV: Positive predictive value. NPV: Negative predictive value.

### Transfer function estimate evaluation

As mentioned in the method section, a frequency sampling filter at the limit of the linear phase frequency region was applied in order to get information about the estimator’s performance, as an additional study. The limit of linear phase varied from around 2 Hz and up to 15 Hz for the patient cohort. Some transfer functions had no linear phase part at all. The filter was applied to both estimates and the invasive ICP gold standard in order to compare signals of equal bandwidth. For these comparisons the error in MWA was notably smaller, but as these results yield little clinical value in our particular clinical application, they are not covered in detail here, but shown in Table 5. The improvement of the MWA prediction, and the varying linear phase limit, indicates that the pressure equalization from the subarachnoid space to the outer ear is only dispersion-free for low frequencies, and that the cochlear aqueduct therefore reproduces the low frequency content of the ICP signal with better accuracy than the high frequency content. The results in Table 5 also reveal that this is highly patient dependent. No significant correlation was found between the improved results and the age or BMI of the patients.

**Table 5:** Transfer function estimate evaluation.

PatID	N 6-sec time windows	Absolute differences between frequency filtered ICP and frequency filtered estimated non-invasive pulsatile ICP			Cut-off frequency (Hz)
		MWA (mmHg)	MWRT (sec)	MWRTC (mmHg/sec)	
1	462	0.7 ± 0.4	0.03 ± 0.02	3.91 ± 1.81	4
2	666	2.3 ± 1.9	0.1 ± 0.04	6.03 ± 4.67	4
3	409	1.4 ± 1.1	0.02 ± 0.02	5.86 ± 4.08	11
4	1044	8.1 ± 5.6	0.03 ± 0.04	20.55 ± 13.12	4
5	682	1.4 ± 0.8	0.04 ± 0.03	4.29 ± 2.31	3
6	321	0.5 ± 0.5	0.01 ± 0.02	2.77 ± 2.61	6
7	906	0.6 ± 0.3	0.01 ± 0.01	2.07 ± 1.25	2
8	557	0.9 ± 0.5	0.05 ± 0.03	3.26 ± 2.06	3
9	624	0.9 ± 0.6	0.03 ± 0.02	2.01 ± 1.89	4
10	771	2.1 ± 1	0.05 ± 0.03	8.19 ± 4.43	3
11	1311	0.7 ± 0.5	0.03 ± 0.03	7.91 ± 7.06	15
12	601	2.6 ± 1	0.03 ± 0.02	13.38 ± 5.24	6
13	1134	1.4 ± 0.3	16.9 ± 3.5	6.64 ± 1.59	6
14	239	1.2 ± 0.9	0.02 ± 0.02	4.29 ± 2.69	5
15	750	2 ± 0.9	0.1 ± 0.04	17.18 ± 6.83	—
16	524	0.9 ± 0.4	0.09 ± 0.05	14.11 ± 6.81	11
17	227	0.6 ± 0.4	0.08 ± 0.03	5.72 ± 2.11	5
18	2460	2 ± 1.7	0.05 ± 0.03	7.28 ± 5.3	4
19	330	0.8 ± 0.7	0.03 ± 0.03	6.73 ± 4.83	10
20	1722	1.8 ± 2	0.02 ± 0.02	6.63 ± 7.1	3
21	265	3.5 ± 2.2	0.02 ± 0.02	12.72 ± 8.72	3
22	498	1.6 ± 0.8	0.08 ± 0.03	17.4 ± 5.65	2
23	559	0.4 ± 0.4	0.05 ± 0.03	1.93 ± 1.39	12
24	347	0.8 ± 0.7	0.01 ± 0.01	3.88 ± 3.34	4
25	2957	1.2 ± 0.8	0.1 ± 0.05	8.73 ± 4.02	5
26	420	0.9 ± 0.8	0.01 ± 0.01	3.34 ± 2.9	4
27	2782	2.3 ± 1	0.07 ± 0.03	9.25 ± 3.31	2
28	791	1.7 ± 1.8	0.02 ± 0.02	4.42 ± 4.75	2
**AVG** ± **STD**		**1**.**6** ± **1**.**5**	**0**.**06** ± **3**.**2**	**7**.**5** ± **5**.**0**	
**SUM**	**24**,**359**				

MWA: Mean Wave Amplitude. MWRT: Mean Wave Rise Time, MWRTC: Mean Wave Rise Time Coefficient.

## Discussion

The present study addresses to which degree measurement of the TMP waveform made non-invasively in the outer ear could estimate the invasively measured ICP waveform. This differs from earlier work in the field, where the goal has been to evaluate absolute, or mean ICP utilizing the stapedial reflex^18,20,22,39,40^. The main observation in this study was that the non-invasive TMP waveforms did not reliably estimate the invasive ICP waveform for the majority of the cohort at the level required for clinical use.

To validate the reliability of the method, we compared the MWA from the invasive ICP signal with the ICP signal estimated from the TMP waveform. From a clinical perspective, the non-invasive MWA was estimated rather satisfactorily in only 4/28 individuals (14%). In these four patients the differences between original and estimated MWA were <1.0 mmHg in >50% of observations, and <0.5 mmHg in >20% of observations (Table 3).

One of the prerequisites of this method is a relatively clear path allowing pressure transfer between the intracranial compartment and the outer ear. For the normal case, where no dehiscence is present, a total of three communication routes exist, namely the vestibular aqueduct, cochlear aqueduct and the internal auditory canal. If there are no irregularities, there is a perfect hydrodynamic relationship between the cerebrospinal fluid pressure and the perilymphatic pressure^32,41,42^. Animal studies show that the cochlear aqueduct is the most prominent route for pressure transfer^43^, but due to the vestibular aqueduct, pressure equalization still happens to some degree also if the cochlear aqueduct is occluded, but if so, with time delay, and with lower magnitude^44,45^. The percentage of patent cochlear aqueducts in humans has been studied by Gopen *et al*.^32^, who found 34% of the cochlear aqueducts to be clearly patent, 7% not to exist or to be completely occluded, and 59% to contain loose connective tissue. From this study alone, a success rate in the range from 34% to 93% would be expected, when assuming that the loose tissue does not completely prevent pressure equalization. Our results are even lower than this.

It should be noted that the result for Patient ID 3 illustrated to the left in Fig. 3, which is representative for the study, is an example of one of the patients where the results from this method were found unsatisfactory. When visually comparing the waveform estimates for Patient ID 3 and Patient ID 9 to the corresponding invasive ICP signal, it is not evident which estimate that performs best. For both patients there is an apparent resemblance in waveform between the nICP estimates and the invasive ICP signal, but when focusing only on the averaged peak-to-peak amplitude, the difference is evidently higher for Patient ID 3 (Table 2). Figure 3 shows that the estimates overshoot for some and undershoot for others, which in sum easily gives an absolute error of more than 1 mmHg. Although MWA is currently the most informative parameter for indicating brain compliance in clinic, and therefore is the measure assessed here, there are other examples in literature where more of the frequency content of the ICP waveform is included in the analysis^14,15^. Given the apparent resemblance in total waveform exemplified in Fig. 3 there are reasons to believe that an analysis focusing on more of the spectral content could give somewhat better results and that the method still might have potential as an initial screening device for ICP.

In the literature, there is some controversy on whether the cochlear aqueduct patency is age dependent. While Gopen *et al*. found no such dependency, an earlier study shows the opposite result^46^. We were not able to find any correlation between age and the ability to estimate MWA from TMP signals in our results. It could be speculated whether age affect the patency of the cochlear aqueduct, and if so, this would affect our results as they are based on a patient cohort with high mean age (54.1 ± 17.8 years).

When visually inspecting the measured TMP signals it seemed as if the mean level of the measurements could fluctuate. Due to the way our study was performed, where the mean pressure was removed, this would not directly affect our spectral estimations, but it indicates that other pressure waves were able to reach the sensor in the outer ear channel. This implies that the sealing was not optimal, and/or that in-body fluctuations affected the measurements. This is substantiated by the fact that the average error in MWA estimation was significantly higher at the end of the measurements compared to the starting point. Measurement uncertainties are therefore most certainly an influencing factor in the presented study, together with the indications that the cochlear aqueduct might result in a dispersive propagation pathway for high frequencies for almost all patients. Unfortunately, this is hard to control as only the external sealing is easily improved.

It is important to note that the method utilized in this study depends on invasively obtained ICP training data, and that therefore also under perfect conditions, the method is of limited use in clinic, as is. The results however, indicate that it could be interesting to investigate alternative ways of estimating patient specific transfer functions. A natural focus for further work is to look into modelling transfer functions in a way not unlike what was proposed by Gao *et al*.^47^, but from the inner ear to the subarachnoid space, which is a different approach to transfer function estimation than what is described in this study. One can imagine a tube reflector system from the perilymphatic space of the inner ear to the intra cranial compartment, where the cochlear aqueduct is modelled as an acoustic tube with a lump that represents the connective tissue. Such a model has already been proposed by Gopen *et al*.^32^. The diameter and length of the tube, as well as maybe even the lump (tissue) within can be found using modern imaging technology. The parameters extracted from images then represent the patient specific adaptiveness of the method^48^. This approach might open up for a risk-free and completely non-invasive ICP monitoring technique that in theory could work for up to the earlier mentioned 34–93% of the patients, under ideal conditions. There are simple tests that can be performed in order to check if the cochlear aqueduct is patent, and hence evaluate at an early stage if such a technique could be relevant for the patient in question. The simplest and most relevant test is the *TMD-test*, which uses the patient’s volume displacement of the tympanic membrane in the outer ear in sitting and supine position. If the volume displacement changes with a given minimum magnitude when the positions changes, the cochlear aqueduct must be patent, as there are known intracranial pressure changes that occur with shifts in posture^40^.

Despite a wide range of approaches over long time, clinically viable non-invasive ICP monitoring is still not a reality. There are techniques that provide promising results^49–51^, but these are currently lacking clinical validation. The importance of ICP level in the case of traumatic brain injury and critical pathologies is well documented, but due to the lack of a good non-invasive monitoring technique, the role played by ICP is unknown in several different less critical pathologies. To create a non-invasive ICP monitoring tool of considerable utility, it must be accurate, non-dangerous, and possible to use for people with relatively little medical expertise. The approach presented here as a possibility for further work is not ideal in this regard, but the risk associated with invasive placement of a pressure probe is removed. In addition, intracranial pressure monitoring through the outer ear, is highlighted by Popovic *et al*. as the technology for non-invasive ICP monitoring that is the most user friendly^5^. However, with traditional tympanic membrane displacement using the stapedial reflex, continuous monitoring is not possible. This changes when looking at the pulsatile pressure and waveform analysis used in this study. The TMP technique utilized here also allows for very comfortable measurements, as there is no exposure of the patients to extreme sound pressure levels, which is necessary in order to trigger the stapedial reflex. It also has the advantage that it can work on people who are on muscle relaxes, which traditional TMD measurements cannot. The potential value for different patient groups if this technique works is therefore significant.

## Conclusion

This study showed that the TMP waveforms measured in the outer ear could not be used to correctly predict the ICP waveform parameter MWA non-invasively. An estimate that might possibly be acceptable in clinic was only observed in 4/28 (14%) individuals. An observation of the study was that the cochlear aqueduct worked as a physical lowpass filter. This reduced the TMP measurement’s ability to reproduce the entire spectral content of the ICP waveform, and hence affected the MWA estimation.

## References

[1] Czosnyka M, Pickard JD (2004). Monitoring and interpretation of intracranial pressure. J. Neurol. Neurosurg. Psychiatry.

[2] Miller JD (1977). Significance of intracranial hypertension in severe head injury. Journal of neurosurgery.

[3] Steiner L, Andrews P (2006). Monitoring the injured brain: ICP and CBF. BJA: British Journal of Anaesthesia.

[4] Binz DD, Toussaint LG, Friedman JA (2009). Hemorrhagic complications of ventriculostomy placement: a meta-analysis. Neurocritical care.

[5] Popovic D, Khoo M, Lee S (2009). Noninvasive monitoring of intracranial pressure. Recent patents on biomedical engineering.

[6] Zhang X (2017). Invasive and noninvasive means of measuring intracranial pressure: a review. Physiological Measurement.

[7] Robba C (2016). Non‐invasive assessment of intracranial pressure. Acta Neurologica Scandinavica.

[8] Czarnik T (2007). Noninvasive measurement of intracranial pressure: is it possible?. Journal of Trauma and Acute Care Surgery.

[9] Czosnyka, M. & Citerio, G. (Springer, 2012).

[10] Löfgren J, Essen CV, Zwetnow NN (1973). The pressure‐volume curve of the cerebrospinal fluid space in dogs. Acta Neurologica Scandinavica.

[11] Avezaat C, Van Eijndhoven J, Wyper D (1979). Cerebrospinal fluid pulse pressure and intracranial volume-pressure relationships. Journal of Neurology, Neurosurgery & Psychiatry.

[12] Takizawa H, Gabra-Sanders T, Miller DJ (1987). Changes in the cerebrospinal fluid pulse wave spectrum associated with raised intracranial pressure. Neurosurgery.

[13] Eide PK (2016). The correlation between pulsatile intracranial pressure and indices of intracranial pressure-volume reserve capacity: results from ventricular infusion testing. Journal of neurosurgery.

[14] Lang EW, Paulat K, Witte C, Zolondz J, Mehdorn HM (2003). Noninvasive intracranial compliance monitoring: technical note and clinical results. Journal of neurosurgery.

[15] Robertson CS (1989). Clinical experience with a continuous monitor of intracranial compliance. Journal of neurosurgery.

[16] Eide P, Czosnyka M, Sorteberg W, Pickard J, Smielewski P (2007). Association between intracranial, arterial pulse pressure amplitudes and cerebral autoregulation in head injury patients. Neurological research.

[17] Wagshul ME, Eide PK, Madsen JR (2011). The pulsating brain: a review of experimental and clinical studies of intracranial pulsatility. Fluids and Barriers of the CNS.

[18] Marchbanks R, Reid A, Martin A, Brightwell A, Bateman D (1987). The effect of raised intracranial pressure on intracochlear fluid pressure: three case studies. British journal of audiology.

[19] Shimbles S, Dodd C, Banister K, Mendelow A, Chambers I (2005). Clinical comparison of tympanic membrane displacement with invasive intracranial pressure measurements. Physiological measurement.

[20] Gwer S (2013). The tympanic membrane displacement analyser for monitoring intracranial pressure in children. Child’s Nervous System..

[21] Raboel P. H., Bartek J., Andresen M., Bellander B. M., Romner B. (2012). Intracranial Pressure Monitoring: Invasive versus Non-Invasive Methods—A Review. Critical Care Research and Practice.

[22] Davids J, Birch A, Marchbanks R (2012). 082 Non-invasive measurements of intracranial pressure: Can Coherent averaging show a tilt-dependent change in the measured Spontaneous Tympanic Membrane Displacement (STMD) signal in healthy volunteers?. J Neurol Neurosurg Psychiatry.

[23] Parker D (1977). Labyrinth and cerebral-spinal fluid pressure changes in guinea pigs and monkeys during simulated zero G. Aviation, space, and environmental medicine.

[24] Beentjes B (1972). The cochlear aqueduct and the pressure of cerebrospinal and endolabyrinthine fluids. Acta oto-laryngologica.

[25] Carlborg B, Densert B, Densert O (1982). Functional patency of the cochlear aqueduct. Annals of Otology, Rhinology & Laryngology.

[26] Carlborg BI, Konrádsson KS, Carlborg AH, Farmer JC, Densert O (1992). Pressure transfer between the perilymph and the cerebrospinal fluid compartments in cats. Otology & Neurotology.

[27] Pranevicius, O., Pranevicius, M., Pranevicius, H., Marcinkevicius, E. & Liebeskind, D. S. (Google Patents, 2012).

[28] Stettin E, Paulat K, Schulz C, Kunz U, Mauer UM (2011). Noninvasive intracranial pressure measurement using infrasonic emissions from the tympanic membrane. Journal of clinical monitoring and computing.

[29] Paulat K, Brucher R, Russell D (2002). Noninvasive monitoring of intracranial pressure and compliance. The Annals of Thoracic Surgery.

[30] Holm S, Eide PK (2009). Impact of sampling rate for time domain analysis of continuous intracranial pressure (ICP) signals. Medical engineering & physics.

[31] Eide PK (2006). A new method for processing of continuous intracranial pressure signals. Medical engineering & physics.

[32] Gopen Q, Rosowski JJ, Merchant SN (1997). Anatomy of the normal human cochlear aqueduct with functional implications. Hearing research.

[33] Evensen KB, O’Rourke M, Prieur F, Holm S, Eide PK (2018). Non-invasive Estimation of the Intracranial Pressure Waveform from the Central Arterial Blood Pressure Waveform in Idiopathic Normal Pressure Hydrocephalus Patients. Scientific reports.

[34] Hayes, M. H. *Statistical digital signal processing and modeling*. 415 (John Wiley & Sons, 1996).

[35] Eide PK, Holm S, Sorteberg W (2012). Simultaneous monitoring of static and dynamic intracranial pressure parameters from two separate sensors in patients with cerebral bleeds: comparison of findings. Biomedical engineering online.

[36] Rabiner L, Gold B, McGonegal C (1970). An approach to the approximation problem for nonrecursive digital filters. IEEE Transactions on Audio and Electroacoustics.

[37] Eide PK, Sorteberg W (2010). Diagnostic intracranial pressure monitoring and surgical management in idiopathic normal pressure hydrocephalus: a 6-year review of 214 patients. Neurosurgery.

[38] Eide PK, Sorteberg W (2016). Outcome of surgery for idiopathic normal pressure hydrocephalus: role of preoperative static and pulsatile intracranial pressure. World neurosurgery.

[39] Marchbanks, R. J. (Google Patents, 1989).

[40] Samuel M, Burge DM, Marchbanks RJ (1998). Tympanic membrane displacement testing in regular assessment of intracranial pressure in eight children with shunted hydrocephalus. Journal of neurosurgery.

[41] Ciuman RR (2009). Communication routes between intracranial spaces and inner ear: function, pathophysiologic importance and relations with inner ear diseases. American journal of otolaryngology.

[42] Hofman R, Segenhout J, Albers F, Wit H (2005). The relationship of the round window membrane to the cochlear aqueduct shown in three-dimensional imaging. Hearing research.

[43] Thalen E, Wit H, Segenhout J, Albers F (2001). Dynamics of inner ear pressure change caused by intracranial pressure manipulation in the guinea pig. Acta oto-laryngologica.

[44] Carlborg BI, Farmer JC (1983). Transmission of cerebrospinal fluid pressure via the cochlear aqueduct and endolymphatic sac. American journal of otolaryngology.

[45] Kishimoto S, Nagahara K, Fisch V, Dillier N (1983). Inner ear pressure measurements. Otolaryngol Clin North Am.

[46] Włodyka J (1978). Studies on cochlear aqueduct patency. Annals of Otology, Rhinology & Laryngology.

[47] Gao M (2016). A Simple Adaptive Transfer Function for Deriving the Central Blood Pressure Waveform from a Radial Blood Pressure Waveform. Scientific reports.

[48] Song Chan Il, Kang Woo Seok, Lee Jeong Hyun, Chung Jong Woo (2016). Diameter of the Medial Side of the Cochlear Aqueduct Is Narrower in Meniere's Disease: A Radiologic Analysis. The Journal of International Advanced Otology.

[49] Levinsky A, Papyan S, Weinberg G, Stadheim T, Eide PK (2016). Non-invasive estimation of static and pulsatile intracranial pressure from transcranial acoustic signals. Medical engineering & physics.

[50] Kashif FM, Verghese GC, Novak V, Czosnyka M, Heldt T (2012). Model-based noninvasive estimation of intracranial pressure from cerebral blood flow velocity and arterial pressure. Science translational medicine..

[51] Koskinen L-OD (2017). Can intracranial pressure be measured non-invasively bedside using a two-depth Doppler-technique?. Journal of clinical monitoring and computing.

